# Tracking the impact of depression in a perspective-taking task

**DOI:** 10.1038/s41598-017-13922-y

**Published:** 2017-11-01

**Authors:** Heather J. Ferguson, James Cane

**Affiliations:** 1 School of Psychology, Keynes College, University of Kent, Canterbury, England; 2 School of Psychology, Politics and Sociology, Canterbury Christchurch University, Canterbury, England

## Abstract

Research has identified impairments in Theory of Mind (ToM) abilities in depressed patients, particularly in relation to tasks involving empathetic responses and belief reasoning. We aimed to build on this research by exploring the relationship between depressed mood and cognitive ToM, specifically visual perspective-taking ability. High and low depressed participants were eye-tracked as they completed a perspective-taking task, in which they followed the instructions of a ‘director’ to move target objects (e.g. a “teapot with spots on”) around a grid, in the presence of a temporarily-ambiguous competitor object (e.g. a “teapot with stars on”). Importantly, some of the objects in the grid were occluded from the director’s (but not the participant’s) view. Results revealed no group-based difference in participants’ ability to use perspective cues to identify the target object. All participants were faster to select the target object when the competitor was only available to the participant, compared to when the competitor was mutually available to the participant and director. Eye-tracking measures supported this pattern, revealing that perspective directed participants’ visual search immediately upon hearing the ambiguous object’s name (e.g. “teapot”). We discuss how these results fit with previous studies that have shown a negative relationship between depression and ToM.

## Introduction

Research has established that people with clinical depression experience significant impairments in everyday social function, which typically persist even after the core symptoms of depression have been relieved^1,2^. These social impairments may in part be due to the relationship between depression and problems interpreting others’ mental states and understanding other people’s actions^3,4^. Socially-relevant abilities such as these come under the umbrella of ‘Theory of Mind’ (ToM), a term that is commonly used to refer to the overarching ability to understand that others have mental states (i.e. thoughts, beliefs, intentions and desires) that may differ from our own. This ability is important for effective social interactions as it allows us to predict other people’s potential actions, and to make relevant and appropriate social responses. Despite numerous studies examining social cognition in depressive disorders, the relationship between the two remains poorly understood. This is partly because most previous studies have focused on affective ToM (i.e. understanding the emotional states of others, typically via facial expressions); depressed individuals are impaired at interpreting others’ affective states and show increased sensitivity to sad emotions (for reviews see^5,6^). In contrast, investigations of the cognitive-social mechanisms that might be affected in depression are much more limited, and none so far have employed sophisticated experimental methods to track ToM use in real-time. Therefore, in this paper we use eye-tracking to examine the degree to which individuals with high and low numbers of depressive symptoms are able to infer other peoples’ visual perspectives online, and use this to guide understanding of verbal instructions in a communication task.

The limited research on depression and function on the cognitive-social domain so far suggests that depressed individuals exhibit impaired ToM performance that is similar, though less severe, to that seen in autistic and psychotic individuals. Specifically, both empathic responses and reasoning about other people’s beliefs are attenuated in those with depression compared to non-depressed controls (e.g^7,8^), with further evidence demonstrating impairments in identifying social faux pas in depressed groups^9,10^. Depression has also been shown to have a particular impact on decoding and reasoning abilities in social situations where contextual information has to be integrated^4^. Interestingly, deficits in ToM abilities have been shown to predict relapse to Major Depression episodes^11^. Therefore, there is growing evidence for a link between depression and impaired cognitive ToM abilities, with the possibility that these deficits may contribute to impairments in social function. However, our understanding of this link is limited since all previous research in this domain has employed ‘offline’ response-based measures of ToM performance. That is, previous research has exclusively used tasks that require participants to answer explicit questions about another person’s mental state (e.g. “why did X do that?”, “how is Y feeling?”), meaning that while peoples’ accuracy at judging others’ mental states is established, nothing is known about the cognitive mechanisms and biases that underlie a particular response. Here, we apply methods from Experimental Psychology to record response times and eye movements in a task that requires participants to take the visual perspective of another person, thus revealing the implicit mentalizing strategies that people deploy in real-time.

Visual perspective-taking ability is couched within ToM and refers to the ability to assimilate *what* another person can see from their perspective (Level 1 perspective taking), or *how* another person can see something (Level 2 perspective taking, see^12^). This ability is particularly useful in social interactions as it allows people to reduce ambiguities, narrowing down the set of objects being referred to, thereby aiding reasoning about another’s intentions and enabling appropriate social responses according to a personal or mutual goal(s) within a particular environment. Among healthy adults, a growing body of research has been conducted to examine the cognitive processes that underlie ToM and perspective-taking ability, with many studies taking advantage of the precision afforded by online measures such as reaction times, eye movements and even electrophysiological responses (e.g^13–16^). A key paradigm that has been developed to examine visual perspective-taking ability is the referential communication task^17^. In this task participants follow the instructions of a ‘director’ to select (“click on the…”) or move (“Move/pick up the…”) target objects (e.g. a ball) around a visual display. The visual display typically consists of a gridded cupboard; some of the objects in the grid are visible to both the director and the participant, but others are occluded from the director’s (but not the participant’s) view. On critical trials, the grid contains a range of objects including a target object (e.g. a toy mouse) and a referentially ambiguous competitor object (e.g. a computer mouse). To examine perspective-taking ability, the competitor object is placed in privileged ground, where it is occluded from the director’s view by a physical barrier. Therefore, to correctly identify the target object from an ambiguous instruction to “move the mouse left”, participants must infer the director’s limited perspective and restrict attention to the mouse in ‘common ground’ (i.e. shared view^18^) while inhibiting access to the competitor in ‘privileged ground’.

This research has demonstrated that the ability to compute another’s visual perspective occurs rapidly (e.g^19–23^), however incorporating this information into a relevant response can be cognitively demanding and is susceptible to errors. Indeed, contrary to the intuition that healthy adults are fully capable ‘mindreaders’, many researchers have reported a delay in selecting (or fixating if using eye movement measures) the perspective-appropriate object when the director and participant hold conflicting knowledge about the available objects, and even explicit errors of selecting a perspective-inappropriate referent (e.g^16,24–27^). This difficulty has been explained in terms of an egocentric or ‘reality’ bias^28^, as people suffer persistent interference from their own knowledge, perhaps even anchoring initial understanding to their own point of view before considering someone else’s^27^.

Using this paradigm in combination with sensitive reaction time and eye movement measures, visual perspective-taking ability has been shown to be influenced by numerous factors including social and cultural relationships^29,30^, executive functions^31–33^, and even affective mood^34^. Specifically, in their study on the effect of mood in perspective-taking, Converse *et al*. (2008) used music and films to induce temporary happy or sad moods in their participants prior to completing a false belief task (Experiment 1) or the referential communication perspective-taking task described above (Experiment 2). Both studies showed that when participants were primed to feel a positive mood they were more prone to egocentric intrusions, showing a decreased propensity to take the other person’s perspective. In contrast, those primed to feel a negative mood were more likely to use the other person’s knowledge to complete the tasks. The authors argue that those in a happy mood rely more on default stereotypes or egocentric knowledge of other peoples’ mental states compared to those in a sad or neutral mood – an idea previously borne out in social judgment studies^35^. However, this pattern is surprising given the previously reviewed evidence for impaired ToM performance in depressed individuals who experience persistent feelings of sadness^36^. Thus, we propose that while a sad mood is a component of most episodes of depression it is likely that an experimentally induced ‘state’ mood is not equable with the prolonged ‘trait’ mood changes that people with depression experience.

In the present study we aimed to build on previous findings by examining how depression impacts the deployment of perspective-taking in real-time. To this end, we employed an eye-tracked version of the referential communication perspective-taking task, and compared performance in two perspective conditions: a ‘Listener Privileged’ condition where the competitor object was only available to the participant, and a control ‘Shared Perspective’ condition where the target and competitor objects were available to both the participant and director condition. Participants were taken from a non-clinical sample, who self-reported high or low numbers of depressive symptoms (measured by the Beck Depression Inventory^37^).

In line with previous research on clinical depression, we predicted that people in the low depression group would successfully use the director’s perspective, so that they experienced less interference from the competitor object when it was in privileged view compared to when it was in shared view. This effect should be manifest in faster response times, and stronger biases to fixate the target object in the Listener Privileged condition compared to the Shared Perspective condition, as participants are less likely to consider the privileged object as a potential referent. In contrast, we predicted that those in the high depression group would show deficits in perspective-taking ability, meaning that they would be delayed in dissociating the target and competitor objects on both the Shared Perspective and Listener Privileged conditions due to difficulty inhibiting the competitor object in privileged ground.

## Method

### Participants

In order to identify participants with high and low numbers of depressive symptoms, we first invited 250 non-psychology students from the University of Kent to complete an online self-report ‘mood questionnaire’- the Beck Depression Inventory (BDI^37^). This questionnaire consists of 21 groups of statements relating to feelings of sadness, pessimism, loss of energy, etc. Participants are instructed to pick out one statement in each group that best describes the way they have been feeling in the past two weeks, including today (e.g. ‘I do not feel sad’, ‘I feel sad much of the time’, ‘I am sad all the time’, or ‘I am so sad or unhappy that I can’t stand it’). Scores can range from 0 to 63, with higher scores indicating more severe depressive symptoms. Using a cutoff of 10 and below to indicate minimal depressive symptoms and 17 and over to indicate moderate or severe depression, we then invited suitable participants to the lab to complete the perspective-taking task.

In total, sixty-two participants (Mage = 19.1; 55 females) took part in the main experiment. They were all native English speakers, had normal or corrected-to-normal vision, and were either paid for participating or received course credits. Thirty-three participants were classified as ‘low depression’ based on their BDI scores (range = 0–10; Mean = 4.7, SD = 3.0) and twenty-nine were classified as ‘high depression’ (range = 17–49; Mean = 25.7, SD = 9.4). The difference in BDI scores between groups was significant, t(60) = 12.19, *p* < 0.001.

### Measures and Design

**Figure 1:**
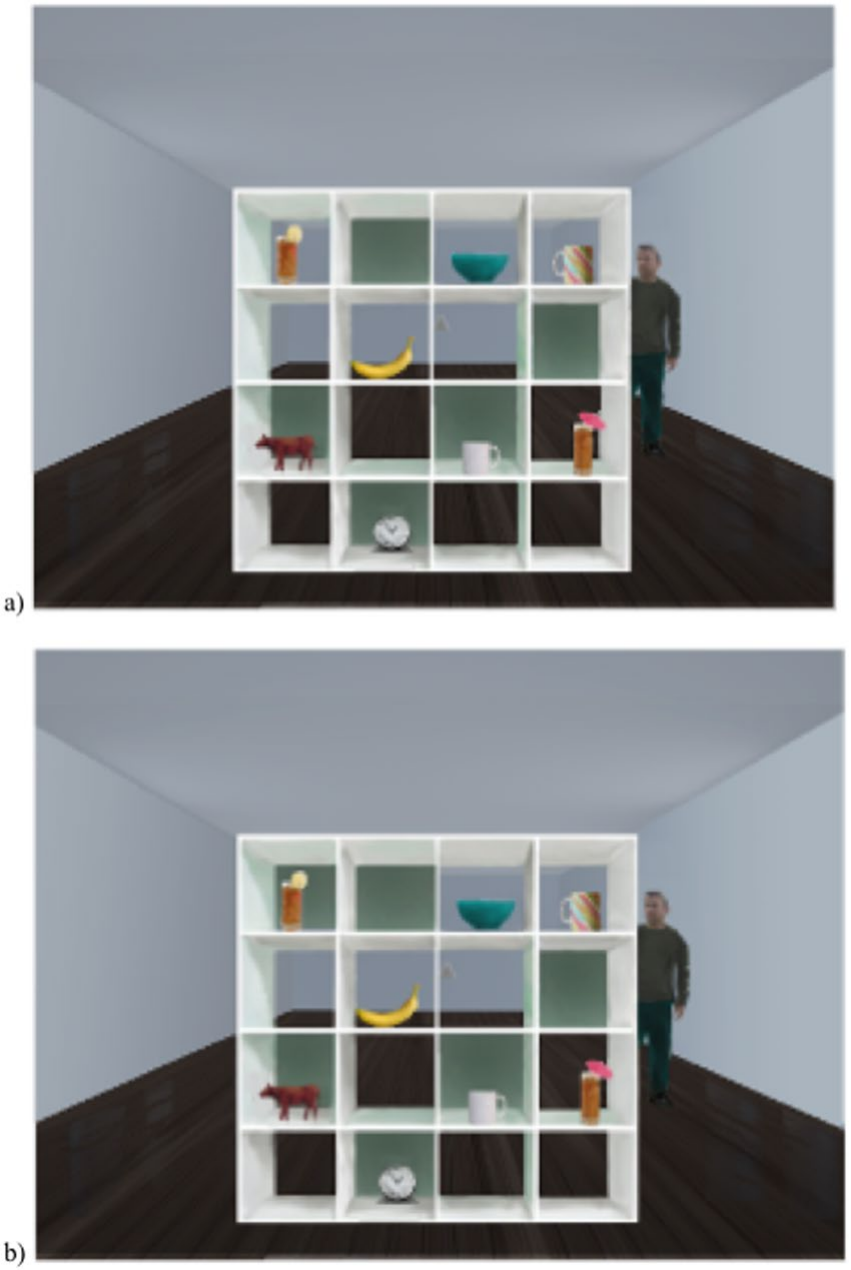
Example stimuli from the referential communication task, to be paired with the instruction, “Move the glass with the umbrella in down”. Panel (a) shows an example scene in the Shared Perspective condition (i.e. both the target and competitor objects are visible to both speaker and participant), and panel (b) shows an example scene in the Listener Privileged condition (i.e. only the target object is visible to both speaker and participant).

Each trial in the perspective-taking task consisted of an image of a room containing a 4 × 4 gridded cupboard with a male avatar standing to the rear right-hand side of the cupboard (see Fig. 1). For both the Listener Privileged and Shared Perspective conditions the backs of five of the spaces within the cupboard were covered so that the contents of these spaces were occluded from the avatar’s view. In total, 26 cupboard configurations were created, two for the practice trials and 24 for the experimental trials (12 Listener Privileged, 12 Shared Perspective). Twelve sets of 7–8 objects were placed into the cupboard spaces. For the Shared Perspective condition each set included a target object (e.g. a glass with an umbrella in) and a competitor object (e.g. a glass with a lemon in), which were both in visual common ground. For the Listener Privileged condition the competitor object was placed in one of the speaker occluded spaces, thus was only visible to the participant. Participants received three instructions per trial, comprising two filler instructions and one critical instruction. Critical instructions consisted of “Move the…” + the target object noun (e.g. ball, shoe, truck) + disambiguating information (e.g. “with the umbrella in”) + a direction (up, down, left, or right). Filler instructions comprised two types, one containing a non-comparative adjective (e.g. “Move the yellow bucket up”) and one not containing an adjective (e.g. “Move the bottle down”). The order of filler and critical instructions was counterbalanced across trials and a new instruction was only given once participants had responded to the previous instruction. The mean onset of the target object noun was 931 msec after the start of the instruction, the mean disambiguating word onset was 1841msec after the start of the instruction, and the mean offset was 2265 msec after the start of the instruction.

Eye movements were recorded using an EyeLink 1000 desktop mounted SR Research eye-tracker, with eye-movements sampled at a frequency of 1000 Hz. The participant’s head was kept immobile with the use of a chin and forehead rest throughout the experiment and only the right eye was tracked. Stimuli were presented on a 19-inch TFT monitor screen with a screen resolution of 1,024~768 pixels 60 cm from the participant. A nine-point calibration sequence was used to calibrate participants’ eye movements and a drift correction check (central fixation point on the screen) was displayed at the start of each trial. The perspective-taking task was delivered and controlled using the Eye-Link Experiment Builder Software (version 1.10.165). Each box on the 4 × 4 grid covered an average visual angle of 4.25° on the horizontal plane and an average visual angle of 5.35° on the vertical plane, dependent on the location of the box. The description on where to move the objects for each trial were delivered in mono sound to participants through headphones covering both ears.

### Procedure

The study was conducted in accordance with British Psychology Society guidelines, and received ethical approval from the University of Kent. Participants sat in front of the monitor and were given instructions on how to complete the referential communication task, using the mouse to move objects one space left, right, up, or down, according to the instructions given by the avatar. Participants were instructed to take the avatars perspective into account throughout; they were shown a single example of the grid from their perspective and from the avatars perspective to ensure they understood that their perspectives differed. Once the participants had indicated they fully understood the instructions, their eye-movements were calibrated and the headphones for the instructions were placed over their ears. Participants then received two practice trials, one replicating a trial from each of the conditions, before moving onto the main set of 24 experimental trials that were randomly presented for each participant. In each trial, participants saw a grid scene for the perspective-taking task, and responded to three instructions to move objects around this grid. The visual locations of objects in the grid were updated in real time as participants moved them. Halfway through the experiment participants were able to take a short break. Once they were ready to continue, eye movements were recalibrated to ensure accuracy and the remaining trials were delivered. Participants gave informed consent prior to participating and were fully debriefed at the end of the experiment.

### Eye movement analyses

Regions of interest (ROIs) were specified around all of the objects within the 4 × 4 cupboard, and the remaining areas were coded as background. Analyses examined looks to the target object (and competitor object) locations in each condition and group. Two fixation measures were calculated. First, we calculated the probability that participants made at least one fixation on the target/competitor object during the ambiguous period, between the onset of the target object’s name (e.g. from the ‘g’ in “glass” for “move the glass with the…”) and the onset of the disambiguating detail (the ‘u’ in “umbrella in”). This analysis period was defined on a trial-by-trial basis according to the absolute onset times for individual words in each item, and lasted for an average of 910 msec (SD: 131; range: 692–1169). There was no difference in the length of this ambiguous period between Listener Privileged (M = 899 msec) and Shared Perspective (M = 922 msec) conditions, *t* < 1. Analysing eye movements prior to the onset of disambiguating information allows us to examine participants’ *anticipatory* eye movements towards objects in the scene- i.e. looks to objects that they expect to be mentioned in the unfolding instruction. The probability of fixating the target object was defined as the sum of fixations to the target object divided by the total number of fixations elsewhere (i.e. all ROIs, including background) on that trial, and the probability of fixating the competitor object was defined as the sum of all fixations to the competitor object divided by the total number of fixations elsewhere on that trial. The second fixation measure examined the latency of first fixations on the target object (i.e. time to first fixate the target object), relative to the onset of the ambiguous noun (e.g. from the ‘g’ in “glass” for “move the glass with the…”). This analysis period was defined on a trial-by-trial basis according to the absolute onset times for individual words in each item, and included any first fixations on the target until participants made a selection response.

## Results

### Accuracy

Mean accuracy was high (98.6%) across all participants, thus no further analyses were conducted on error rates for the director task. These near ceiling responses show that all participants were successfully able to complete the task (since ambiguity was always resolved at sentence end, e.g. “umbrella in”), and support our focus on the implicit measures that underlie processing prior to object selection. All trials with selection errors were removed from subsequent analyses. All data is publically available at https://osf.io/2vdzc/?view_only = 501bc4a276804957899acf3aa4ff17b8.

### Response times

**Figure 2:**
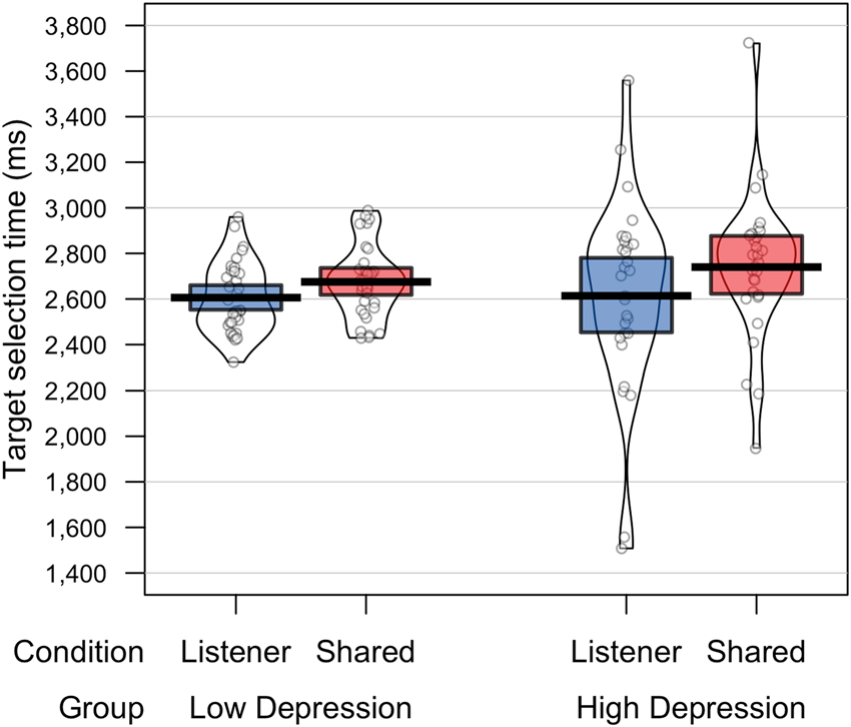
Target selection response times on the perspective-taking task, for each condition and depression group.

Outlier data points were detected and removed from the target selection response times using a cutoff of 2.5 standard deviations from the mean of each group, which removed 1.78% of the data. The resulting reaction times were entered into a 2 (Group: Low *vs*. high depression) × 2 (Condition: Shared Perspective *vs*. Listener Privileged) mixed model ANOVA with Group as the between subjects factor and Condition as the within subject factor. The data are plotted in Fig. 2, showing raw data points, a horizontal line reflecting the group/condition mean, a bean showing smoothed density, and a rectangle representing the Bayesian highest density interval. Statistical analysis revealed a significant main effect of Condition, *F*(1,60) = 18.59, *p* < 0.001, _p_η² = 0.24, with significantly longer reactions times in the Shared Perspective condition (*M* = 2708 ms) compared to the Listener Privileged condition (*M* = 2610 ms). There was no significant main effect of Group (*F*(1,60) = 0.28, *p* = 0.6) or a significant interaction between Condition and Group (*F*(1,60) = 1.59, *p* = 0.21).

In addition, a bivariate correlation analysis between participants’ BDI scores and their perspective-taking score (calculated as the RT difference between responses in the Listener Privileged and Shared Perspective conditions) showed no significant correlation (r(62) = 0.09, *p* = 0.49).

### Probability of fixating

**Figure 3:**
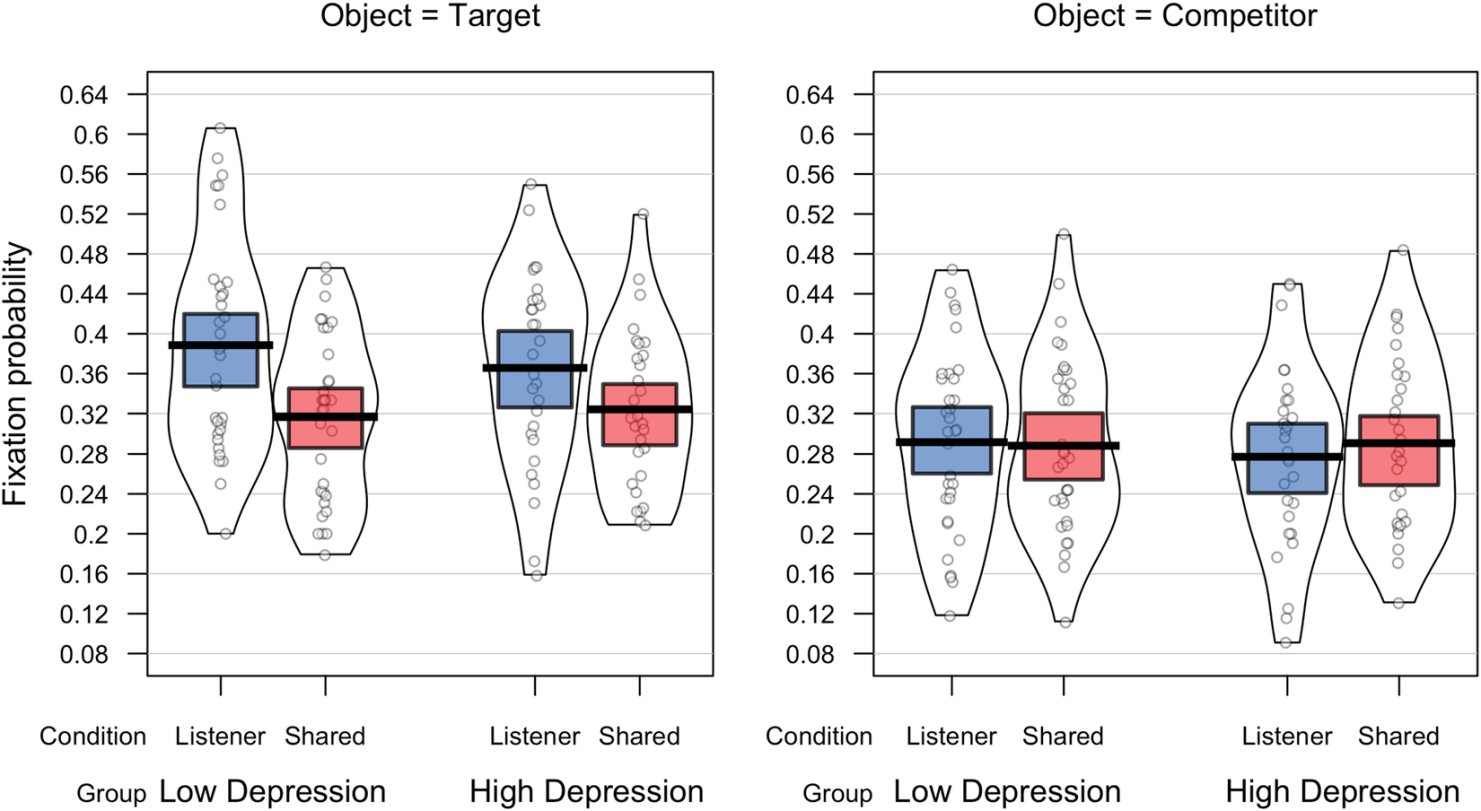
Probability of fixating the competitor object and the target object for each condition and depression group.

The mean probabilities of making an anticipatory fixation on the target object and competitor object for each condition and depression group are plotted in Fig. 3. Data was analysed using a 2 (Group: High *vs*. Low depression) × 2 (Condition: Shared Perspective *vs*. Listener Privileged) × 2 (Object: Target *vs*. Competitor) mixed-model ANOVA. Analyses revealed a significantly higher probability of fixating the target object compared to the competitor object (0.35 *vs*. 0.29; *F*(1,60) = 16.86, *p* < 0.001, _p_η² = 0.22), however this effect was qualified by a significant interaction between Object and Condition, *F*(1,60) = 6.51, *p* = 0.01, _p_η² = 0.1. Follow-up analyses showed that participants were equally likely to fixate the competitor object in the Shared Perspective and Listener Privileged conditions, *t* < 1, but were significantly more likely to make anticipatory fixations on the target object in the Listener Privileged condition compared to the Shared Perspective condition, *t*(61) = 3.78, *p* < 0.001. In addition, participants were significantly more likely to fixate the target object than the competitor object in the Listener Privileged condition, *t*(61) = 4.5, *p* < 0.001, but showed no object preference in the Shared Perspective condition, *t* = 1.4. Despite a numerically larger effect of condition on fixations to target in the low depression group than the high depression group, none of the interactions involving group reached significance, all *F*s < 1.

A bivariate correlation analysis between participants’ BDI scores and their perspective-taking score (calculated as the difference in the probability of fixating the target between the Listener Privileged and Shared Perspective conditions) showed no significant correlation (r(62) = −0.14, *p* = 0.2).

### Latency of first fixation to target

**Figure 4:**
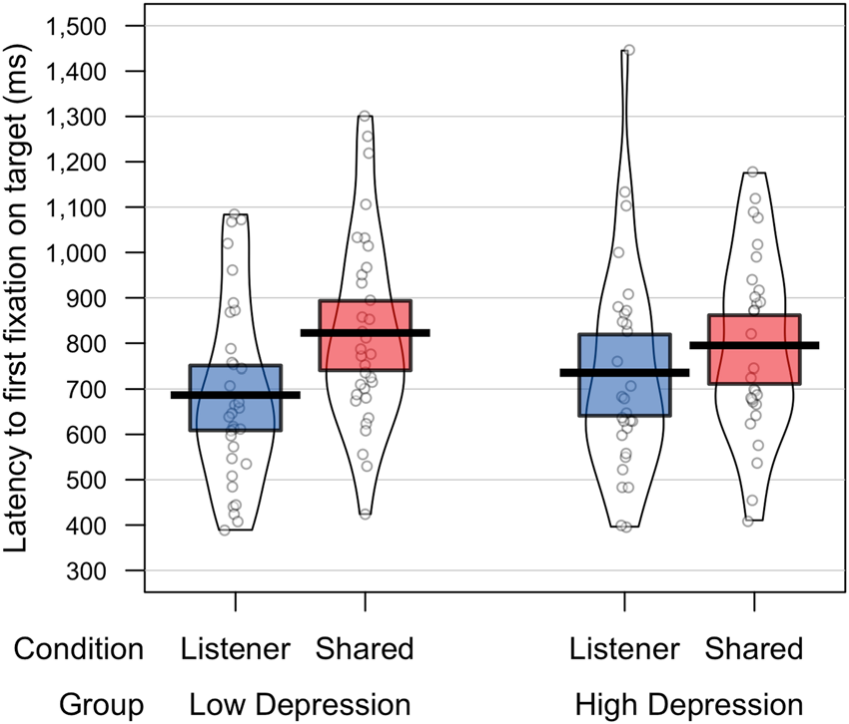
Mean latency to first fixate the target object for each condition and depression group.

The mean latency to first fixate the target object for each condition and depression group are plotted in Fig. 4. Data was analysed using a 2 (Group: High *vs*. Low depression) × 2 (Condition: Shared Perspective *vs*. Listener Privileged) mixed-model ANOVA. Analyses showed that overall, participants were significantly faster to first fixate the target object in the Listener Privileged condition compared to the Shared Perspective condition (711ms *vs*. 809ms; *F*(1,60) = 9.61, *p* < 0.005, _p_η² = 0.14). Despite a numerically larger effect of condition in the low depression group than the high depression group, neither the main effect of Group (*F* < 0.1) or the interaction between Group and Condition (*F* < 1.46, *p* > 0.23) was significant.

A bivariate correlation analysis between participants’ BDI scores and their perspective-taking score (calculated as the latency difference between first fixations to target in the Listener Privileged and Shared Perspective conditions) showed no significant correlation (r(62) = −0.19, *p* = 0.15).

## General Discussion

The present study sought to identify the impact of depression on ToM ability, specifically in the social-cognitive domain of perspective-taking. Eye movements and behavioural responses were recorded whilst participants completed a communication task where they had to use perspective cues to resolve a referential ambiguity (e.g. identifying the correct ‘glass’ to move when more than one glass was visually present). The effects of perspective-taking were compared between participants who self-reported a high and low number of depressive symptoms in two perspective conditions: a ‘Listener Privileged’ condition, where a competitor object was only available to the participant, and a ‘Shared Perspective’ condition, where the competitor object was mutually available to the participant and avatar.

In both the high and low depression groups, participants successfully used perspective cues to disambiguate the target object from the competitor object. This effect was evidenced by faster reaction times to select the target object in the Listener Privileged condition compared to the Shared Perspective condition. Thus, at the point of selection, participants were able to override their own egocentric biases to accommodate another person’s perspective. Further evidence was shown in the real-time eye-tracking data, which revealed that immediately upon hearing the ambiguous object’s name (e.g. “glass”), participants in both groups showed a stronger bias to fixate the target object when the competitor object was in privileged versus shared view, and looked faster towards the target object when perspective allowed them to inhibit the competitor object (i.e. when this competitor was hidden from the director). Nevertheless, this eye-tracking data also showed that participants were equally likely to fixate the competitor object when it was mutually available (i.e. in the Shared Perspective) and when it was hidden from the speaker’s perspective (Listener Privileged), suggesting that although participants *preferred* to fixate the mutually available target in the Listener Privileged condition, they also experienced interference from the hidden competitor object in their own egocentric view. These results are therefore consistent with the patterns found in previous studies that have examined the cognitive processes that underlie ToM and perspective-taking ability in healthy adults (e.g.^17,19,20,23,32^). Importantly, the fact that group did not modulate these effects suggests that depression had little impact on the speed with which individuals were able to use perspective cues to interpret ambiguous communication, and that participants with high and low numbers of depressive symptoms experienced comparable levels of egocentric intrusions.

The finding of preserved cognitive ToM in depressed individuals is interesting because it contrasts with previous research that has shown deficits in ToM ability in depression. Specifically, a relatively robust relationship has been found between depressive symptoms and affective ToM (see^5,6^ for reviews), though the relationship with cognitive ToM is less stable, with some studies reporting impairments (e.g.^7–10^), and others showing little difference between depressed and non-depressed groups (e.g.^4,7,38^). As such, it is possible that the negative impact of depression on ToM is limited to affective and social aspects of cognition, meaning that higher-level cognitive processes are intact (see^39^). The current task required participants to adopt the director’s visual perspective to correctly interpret their instructions, thus the demands on mental state decoding (inferring their thoughts, beliefs, desires, intentions etc) were relatively low. In addition, it is possible that the use of an on-screen, rather than live director, may have reduced participants’ social engagement with the task, and thus masked some of the ToM difficulties that people with depression experience in everyday life. Using an avatar and pre-recorded speech in the current task eliminated variations in verbal and non-verbal behaviours that are common in live conversation, and was based on previous studies (e.g.^32,40,41^). However, future studies should aim to increase the ecological validity of this task to examine whether people with depression exhibit impaired perspective-taking performance on this task when interacting with a real person.

The results are therefore more in line with Converse *et al*.’s^34^ study that employed a similar communication task to ours, and showed comparable ToM use when participants were primed to feel sad and neutral moods (though impaired ToM when they were primed to feel happy). It is also consistent with research that has shown intact cognitive processing in a sad mood^42^, despite significant executive dysfunctions in major depression (for reviews, see^43,44^), since these general executive skills are known to play a vital role in successful ToM^31–33^. Thus, we infer that our high depression sample had preserved executive skills (i.e. inhibitory control and working memory), which allowed them to resist excessive interference from the perspective inappropriate competitor object. Correspondingly, it is important to consider whether the lack of impairments seen in the current study relate to the severity of depressive symptoms among our depressed participants, and whether the increased BDI scores are more reflective of a low mood than cognition-altering clinical depression. A recent meta-analysis of ToM in major depression has revealed that the magnitude of ToM impairments in depressive disorders is significantly related to the severity of depressive symptoms (ref.^45^ see also^46^). The participants tested here were from a non-clinical sample, meaning that they were not recruited on the basis of a formal diagnosis of major depression. Instead, we assessed depressive symptoms using the BDI, and classified participants into statistically different high and low groups. Though none of the interactions with group reached significance in the current study, the eye-tracking data leave open the possibility that people with high levels of depression might experience more interference from their own egocentric perspective than people without depression; high depression participants showed a numerically smaller and slower bias to fixate the target object in the Listener Privileged condition. Indeed, while our correlation analyses between participants’ BDI scores and their perspective-taking ability were not significant, the eye-tracking data showed a trend for this pattern, with target bias decreasing and latency to fixate the target increasing as depression severity increased. As such, while the null effects of group found in the current study suggest that perspective-taking ability is not influenced by sub-clinical levels of depression, future research should investigate whether significant difficulties would emerge on this socio-cognitive dimension when the severity of depression is increased.

More broadly, the present study links to previous findings showing that cognitive distortions linked to depression may impact cognitive tasks by modulating negative expectations of performance, rather than depression directly impacting the underlying cognitive processes. Cognitive distortions and ‘learned helplessness’ are prevalent in depression and can lead to both negative expectations of the future, and negative appraisals of previous performance^47–49^, however the impact of these negative ruminations on cognitive tasks remains under debate. Some studies on patients with a mild head injury have shown no effect of negative expectations on performance^50^, and others have shown impairments in a range of cognitive tasks when patients were explicitly reminded that their diagnosis might lead to poorer cognitive performance^51,52^, independent of any co-morbid depression diagnosis. Thus, whilst the impact of negative expectations on cognitive tasks remains inconclusive, this line of research suggests that depression itself may not impair ToM performance, but that difficulties may emerge when task goals are more explicit (e.g. judging others’ emotions using the Mind in the Eyes task, as in previous research) and participants hold existing negative expectations of their performance. In contrast, when a task manipulates more subtle variables between conditions and measures implicit performance using continuous measures (e.g. the shared *vs*. listener conditions and response latencies/eye movement measures used here), participants will be less aware of what is being tested and have no prior expectations about performance meaning that performance is unaffected. Further research is needed to identify whether and how the negative expectations and distorted cognitions that are hallmark of depression influence performance on different ToM tasks.

Finally, we note that a complex relationship between emotion and perspective-taking has begun to emerge in recent research, with happy and angry emotions increasing egocentric biases, guilt and sadness enhancing inferences about others’ perspectives, and shame impairing participants’ ability to handle conflicting perspectives without influencing attention allocation^34,53–55^. In addition, a recent study has shown individual differences in perspective-taking ability (including relative biases from self and other perspectives) that are modulated by emotional states, including sadness and happiness^56^. Research has identified that depression can be differentiated as a personality trait and also as an emotional state, and it is the latter which is most associated with non-clinical samples (see^57–59^). However, the BDI is more likely to be a measure of trait depression, particularly in female respondents^58^, meaning that it’s unclear to what extent the depression measured in this study represents a trait or state level of depression. Since depression involves a mixture of many different ‘state’ emotions (often specific to an individual and changing day-to-day), which influence perspective-taking in different ways, future research should aim to understand how these complex combinations of state and trait emotions interact to influence ToM use over time.

In conclusion, the current study demonstrates that people with high and low levels of depression show a comparable ability to rapidly integrate another person’s visual perspective, and use this in real-time to interpret their verbal descriptions. This suggests that depression does not influence the high-level cognitive ability to inhibit one’s own egocentric point of view and infer another person’s view. These findings support a model of the ToM-depression relationship whereby deficits are specific to affective and social domains, leaving some cognitive aspects of ToM intact.
